# Associations between iron markers with hemoglobin and outcomes in peritoneal dialysis patients: results from the PDTAP study

**DOI:** 10.1093/ckj/sfae427

**Published:** 2024-12-30

**Authors:** Zi Wang, Guiling Liu, Li Hao, Shaomei Li, Huaying Pei, Jinghong Zhao, Ying Zhang, Zibo Xiong, Yumei Liao, Ying Li, Qiongzhen Lin, Wenbo Hu, Yulin Li, Zhaoxia Zheng, Liping Duan, Gang Fu, Shanshan Guo, Beiru Zhang, Rui Yu, Fuyun Sun, Xiaoying Ma, Zhanzheng Zhao, Jing Xiao, Yulan Shen, Yong Zhang, Xuanyi Du, Tianrong Ji, Caili Wang, Lirong Deng, Yingli Yue, Shanshan Chen, Zhigang Ma, Yingping Li, Li Zuo, Huiping Zhao, Xianchao Zhang, Xuejian Wang, Yirong Liu, Xinying Gao, Xiaoli Chen, Hongyi Li, Shutong Du, Cui Zhao, Zhonggao Xu, Li Zhang, Hongyu Chen, Li Li, Lihua Wang, Yan Yan, Yingchun Ma, Yuanyuan Wei, Jingwei Zhou, Yan Li, Jie Dong

**Affiliations:** Renal Division, Department of Medicine, Peking University First Hospital; Institute of Nephrology, Peking University; Key Laboratory of Renal Disease, Ministry of Health; Key Laboratory of Renal Disease, Ministry of Education, Beijing, China; Renal Division, Department of Medicine, The Second Affiliated Hospital of Anhui Medical University, Anhui, China; Renal Division, Department of Medicine, The Second Affiliated Hospital of Anhui Medical University, Anhui, China; Renal Division, Department of Medicine, The Second Hospital of Hebei Medical University, Hebei, China; Renal Division, Department of Medicine, The Second Hospital of Hebei Medical University, Hebei, China; Department of Nephrology, the Key Laboratory for the Prevention and Treatment of Chronic Kidney Disease of Chongqing, Chongqing Clinical Research Center of Kidney and Urology Diseases, Xinqiao Hospital, Army Medical University (Third Military Medical University), Chongqing, China; Department of Nephrology, the Key Laboratory for the Prevention and Treatment of Chronic Kidney Disease of Chongqing, Chongqing Clinical Research Center of Kidney and Urology Diseases, Xinqiao Hospital, Army Medical University (Third Military Medical University), Chongqing, China; Renal Division, Department of Medicine, Peking University Shenzhen Hospital, Guangdong, China; Renal Division, Department of Medicine, Peking University Shenzhen Hospital, Guangdong, China; Renal Division, Department of Medicine, The Third Hospital of Hebei Medical University, Hebei, China; Renal Division, Department of Medicine, The Third Hospital of Hebei Medical University, Hebei, China; Renal Division, Department of Medicine, People's Hospital of Qinghai Province, Qinghai, China; Renal Division, Department of Medicine, People's Hospital of Qinghai Province, Qinghai, China; Renal Division, Department of Medicine, Handan Central Hospital, Hebei, China; Renal Division, Department of Medicine, Handan Central Hospital, Hebei, China; Renal Division, Department of Medicine, Peking Haidian Hospital, Beijing, China; Renal Division, Department of Medicine, Peking Haidian Hospital, Beijing, China; Department of Nephrology, Shengjing Hospital of China Medical University, Shenyang, Liaoning, China; Department of Nephrology, Shengjing Hospital of China Medical University, Shenyang, Liaoning, China; Renal Division, Department of Medicine, Cangzhou Central Hospital, Hebei, China; Renal Division, Department of Medicine, Cangzhou Central Hospital, Hebei, China; Renal Division, Department of Medicine, The First Affiliated Hospital of Zhengzhou University, Henan, China; Renal Division, Department of Medicine, The First Affiliated Hospital of Zhengzhou University, Henan, China; Renal Division, Department of Medicine, Beijing Miyun District Hospital, Beijing, China; Renal Division, Department of Medicine, Beijing Miyun District Hospital, Beijing, China; Renal Division, Department of Medicine, The Second Affiliated Hospital of Harbin Medical University, Heilongjiang, China; Renal Division, Department of Medicine, The Second Affiliated Hospital of Harbin Medical University, Heilongjiang, China; Renal Division, Department of Medicine, The First Affiliated Hospital of BaoTou Medical College, Neimenggu, China; Renal Division, Department of Medicine, The First Affiliated Hospital of BaoTou Medical College, Neimenggu, China; Renal Division, Department of Medicine, People's Hospital of Langfang, Hebei, China; Renal Division, Department of Medicine, People's Hospital of Langfang, Hebei, China; Renal Division, Department of Medicine, People's Hospital of Gansu, Gansu, China; Renal Division, Department of Medicine, People's Hospital of Gansu, Gansu, China; Renal Division, Department of Medicine, Peking University People's Hospital, Beijing, China; Renal Division, Department of Medicine, Peking University People's Hospital, Beijing, China; Renal Division, Department of Medicine, Pingdingshan First People's Hospital, Henan, China; Renal Division, Department of Medicine, Pingdingshan First People's Hospital, Henan, China; Renal Division, Department of Medicine, The First People's Hospital of Xining, Qinghai, China; Renal Division, Department of Medicine, The First People's Hospital of Xining, Qinghai, China; Renal Division, Department of Medicine, Taiyuan Central Hospital, Shanxi, China; Renal Division, Department of Medicine, Taiyuan Central Hospital, Shanxi, China; Renal Division, Department of Medicine, Cangzhou People's Hospital, Hebei, China; Renal Division, Department of Medicine, Cangzhou People's Hospital, Hebei, China; Renal Division, Department of Medicine, First Hospital of Jilin University, Jilin, China; Renal Division, Department of Medicine, First Hospital of Jilin University, Jilin, China; Renal Division, Department of Medicine, The People's Hospital of Chuxiong Yi Autonomous Prefecture, Yunnan, China; Renal Division, Department of Medicine, The People's Hospital of Chuxiong Yi Autonomous Prefecture, Yunnan, China; Renal Division, Department of Medicine, The Second Hospital of Shanxi Medical University, Shanxi, China; Renal Division, Department of Medicine, The Second Hospital of Shanxi Medical University, Shanxi, China; Renal Division, Department of Medicine, China Rehabilitation Research Center, Beijing Boai Hospital, Beijing, China; Renal Division, Department of Medicine, China Rehabilitation Research Center, Beijing Boai Hospital, Beijing, China; Renal Division, Department of Medicine, Beijing Dongzhimen Hospital, Beijing, China; Renal Division, Department of Medicine, Beijing Dongzhimen Hospital, Beijing, China; Renal Division, Department of Medicine, Peking University First Hospital; Institute of Nephrology, Peking University; Key Laboratory of Renal Disease, Ministry of Health; Key Laboratory of Renal Disease, Ministry of Education, Beijing, China

**Keywords:** ferritin, PDTAP, peritoneal dialysis, TSAT

## Abstract

**Background and hypothesis:**

Iron metabolism markers, such as transferrin saturation (TSAT) and ferritin, are crucial in anemia management in patients with CKD and those undergoing dialysis, yet optimal levels remain unelucidated.

**Methods:**

We conducted a prospective multicenter cohort study using data from the nationwide Peritoneal Dialysis Telemedicine-based Management Platform (PDTAP) to analyze TSAT, ferritin, and hemoglobin (Hb) levels, and their associations with mortality in the peritoneal dialysis (PD) population.

**Results:**

Our study included 4429 PD patients, analyzing data through restricted cubic splines and Cox regression models, adjusted for multiple confounders. Non-linear associations between Hb levels and TSAT/ferritin were observed. Hb levels increased with TSAT up to 40%, then plateaued, whereas ferritin levels increased with the decline of Hb. Further, ferritin levels above 200 ng/mL were independently linked to increased mortality risk [hazard ratio (HR) 1.207, 95% confidence interval (CI) 1.134–1.286], with this effect decreasing as high-sensitivity C-reactive protein levels rose. This risk was notably significant in patients with a history of cardiovascular disease. A ferritin/Hb ratio >2 was associated with increased risk of mortality after adjusting for demographic, nutritional factors and erythropoiesis agents (HR 1.219, 95% CI 1.144–1.299). The ferritin/Hb ratio demonstrated superior predictive ability for iron responsiveness compared with ferritin alone.

**Conclusion:**

Serum ferritin level exceeding 200 ng/mL was indepently associated with a higher risk of martality in Chinese PD population. Monitoring the ferritin/Hb ratio may help assess the relative iron content in the body and provide reference for iron supplementation among patients undergoing PD.

## INTRODUCTION

Anemia is a prevalent complication among individuals with CKD, significantly elevating the risks of morbidity and mortality and reducing quality of life for these patients [1, 2]. Iron metabolism plays a critical role in the management of anemia in patients with CKD, including those undergoing peritoneal dialysis (PD) and hemodialysis (HD). High doses of erythropoiesis-stimulating agents (ESAs) have been linked to increased mortality in dialysis patients [3, 4]. Iron supplementation is used to reduce the need for high doses of ESAs by balancing the benefits of minimizing blood transfusions and managing anemia-related symptoms against potential risks to patients [5]. However, excessively high levels of iron markers may also increase the risk of infections and cardiovascular complications, underscoring the need for careful monitoring and adjustment of iron therapy [6]. Currently, most guidelines recommend iron supplementation if TSAT is below 20% and serum ferritin levels are under 100 ng/mL. However, upper limits differ, with guidelines in Western countries typically suggesting TSAT less than 30%–50% and ferritin less than 500–800 ng/mL [5, 7–9], in contrast to more conservative Japanese guidelines [10].

The iron supplement and iron status in PD patients differ from those in patients on HD to some extent. Although PD patients typically experience less routine blood loss and may therefore require lower doses of iron supplementation [11], gastrointestinal side effects from oral iron supplements and the inconvenience of intravenous administration can contribute to poor adherence to iron therapy. According to the Dialysis Outcomes and Practice Patterns Study (PDOPPS), 16%–23% of patients undergoing PD did not achieve a hemoglobin (Hb) level of 100 g/L, and ferritin and TSAT levels varied significantly across countries [12]. To date, studies on the importance of maintaining specific ranges of ferritin and TSAT to effectively manage anemia and reduce the risk of adverse outcomes are performed mostly in CKD [13, 14] and HD [15, 16] patients. The practice patterns of iron supplementation and the appropriate ranges of ferritin and TSAT among PD patients have not been thoroughly investigated yet.

In the current study, we aimed to explore the quantitative relationship curves between TSAT, ferritin, and Hb based on a national-level dataset from the PDTAP study. We would further characterize the association between TSAT and ferritin levels and mortality, taking potential confounders into account.

## MATERIALS AND METHODS

### Study design and participants

This is a subsidiary study of the Peritoneal Dialysis Telemedicine-based Management Platform (PDTAP) study, a nationwide multicenter, prospective study. Center enrollment, participant eligibility, and enrollment details have been published in a previous study [17]. Each participant provided informed consent by signing a form subsequent to the respective center obtaining approval from the ethics board. The study adhered to the principles outlined in the Declaration of Helsinki. As a condition of their consent, participants authorized the use of their individual data in future research endeavors.

### Study population

The healthcare system in China operates under a mixed public–private model with universal health coverage provided through various insurance schemes, including medical insurance for urban residents and the new rural cooperative medical care system. The system aims to provide broad access to healthcare services for all citizens, though the level of coverage and access can vary by region. Center enrollment, participant eligibility, and enrollment details have been published in a previous study [17]. In the current study, we screened all incident and prevalent PD patients between 1 June 2016 and 30 April 2019 who were aged over 14 years and had undergone PD treatment for more than 3 months due to end-stage kidney disease. The database follow-up information was updated until 31 December 2020. Patients who lacked data on TSAT and ferritin were excluded from the current analysis. We also excluded patients with inadequate iron and ESA information (Supplementary Fig. S1).

### Clinical variables

In the study, PD staff collected comprehensive data including demographic details, primary diseases, comorbidities, and clinical outcomes. Data acquisition methods included direct exports from hospital laboratory information management systems and manual entries by PD staff. The baseline values were determined by averaging the laboratory measurements taken during the first 3 months after enrollment, with subsequent updates for laboratory variables every 3 months and dialysis adequacy assessments biannually. TSAT was calculated as the ratio of serum iron and total iron-binding capacity multiplied by 100.

### Drug information

The protocols for ESA and iron supplementation are standardized across participating hospitals in the PDTAP study. Recombinant human erythropoietin-α (rHuEPO-α) is the sole ESA utilized, with dosing adjusted for body weight and clinical response, expressed as units (U) per kilogram per week. Iron supplementation is administered based on serum ferritin and TSAT levels, with doses calculated in milligrams per day. These protocols align with the KDIGO guidelines, which recommend individualized anemia management to maintain Hb levels within target ranges while minimizing risks associated with overtreatment. At each visit, patient medication information was recorded. Doses of specific drugs were calculated using measurements taken over the preceding 3 months.

### Reference anemia guidelines

The PDTAP study does not enforce a uniform protocol across all participating hospitals; however, all participating hospitals adhere to the KDIGO Clinical Practice Guidelines for Anemia in Chronic Kidney Disease, which suggest initiating ESA therapy to prevent Hb levels from falling below 90 g/L and avoiding levels above 115 g/L. These guidelines inform the management of anemia in PD patients, including indications for PD initiation and adjustments in prescription based on clinical parameters.

### Outcome

All patients were followed up until they were transferred to HD, received a renal transplantation, died, were lost to follow-up, or until the end of the study (31 December 2020). The outcome of the current study was all-cause death.

### Statistical analyses

We present patient baseline characteristics and treatments as means ± standard deviations, medians with interquartile ranges, or percentages, as appropriate. Restricted cubic splines were performed to illustrate the association between Hb and iron markers (ferritin and TSAT), with adjustment for confounders including age, gender, Charlson score, BMI [18], serum albumin[19], intact parathyroid hormone (iPTH) [20], hypersensitive C-reactive protein (hsCRP) [19], elemental iron and ESA doses.

In the current study we introduced the ferritin/Hb ratio to provide a more comprehensive assessment of iron metabolism and erythropoiesis by combining two complementary markers: ferritin, which reflects iron storage, and Hb, which indicates the body’s ability to utilize iron for red blood cell production. While ferritin can act as an acute-phase reactant and may be elevated in the presence of inflammation, the ferritin/Hb ratio may enhance the predictive effect for poor prognosis by integrating these markers. This approach offers insights into the balance between iron storage and utilization, potentially aiding in the differentiation of inflammation-driven ferritin elevation from true iron deficiency.

We employed restricted cubic splines to depict the association of iron markers (ferritin, TSAT) and the ferritin/Hb ratio with mortality across four models of increasing adjustment: Model 1: unadjusted; Model 2: adjusted for age and gender; Model 3: Model 2 plus additional adjustment for cardiovascular disease (CVD) history, serum albumin, serum total cholesterol, and hsCRP, as illustrated by our previous study [21]; Model 4: Model 3 plus additional adjustment for doses of elemental iron and ESA.

Based on the findings from the fully adjusted Model 4, which demonstrated a significantly increased risk of mortality in patients with ferritin >200 ng/mL and a ferritin/Hb ratio >2, we further investigated the potential interaction between these two markers and systemic inflammation, as indicated by hsCRP levels. To do this, we stratified the population into tertiles based on hsCRP levels and performed Cox regression analyses with mortality as the outcome. The results of these interaction analyses are presented in a forest plot, showing hazard ratios (HR) and 95% confidence intervals (CI) across the three hsCRP tertiles (Fig. 3).

Sensitivity analyses were performed to test the association between ferritin levels and risk of death according to different levels of Hb (<110 vs ≥110 g/L), whether or not there was a CVD history. To address potential variability across different centers, we conducted a sensitivity analysis to assess the center effect on the association of ferritin >200 ng/mL and ferritin/Hb ratio >2 with mortality. For this analysis, we included center as a random effect in a mixed-effects Cox regression model to estimate the HR and 95% CI for both variables (Supplementary Fig. S2).

To evaluate the predictive value of the ferritin/Hb ratio compared with ferritin alone for responsiveness to iron therapy and mortality risk, we conducted a receiver operating characteristic (ROC) curve analysis. Responsiveness to iron therapy was defined as having a baseline Hb concentration between 110 and 120 g/L, with either maintenance of this range or an increase of 10 g/L after 6 months. We calculated the area under the curve (AUC) for both ferritin and the ferritin/Hb ratio to assess their predictive performance. Statistical comparisons of AUCs were performed using DeLong's test to determine any significant differences.

Missing data were handled using multiple imputation by chained equations (MICE) with the predictive mean matching (PMM) method. The imputation was performed on variables including demographic, clinical, and laboratory data. Five imputed datasets were generated (*m* = 5), and the results were pooled for subsequent Cox regression analyses. The proportion of patients with missing data was <10% for all imputed covariates, with the exception of hsCRP (30% missing), total *Kt*/*V* urea (22% missing), and weekly ESA dose (23% missing).

Statistical analyses were performed using R version 4.3.1 (R Foundation for Statistical Computing, Vienna, Austria). A two-tailed *P*-value <.05 was considered statistically significant.

## RESULTS

### Baseline clinical characteristics

A total of 4429 PD patients from 27 medical centers enrolled in PDTAP were included in the current study (Supplementary Fig. S1). At baseline, the mean Hb level was 103.5 ± 19.9 g/L and the serum albumin level averaged 35.51 ± 5.31 g/L. The median hsCRP level was 2.61 (IQR 0.81–7.29) mg/L . The median levels were 25.9 (19.7–33.9) % for TSAT and 191.0 (86.7–376.0) ng/mL for ferritin. There were 2327 (52.5%) patients received elemental iron therapy, with a mean dose of 79 ± 67 mg/day and 3372 (76.1%) patients were treated with 125 (86–167) U/kg/week of rHuEPO-α (Table 1).

**Table 1:** Characteristics of the study subjects.

	Total population *N* = 4429	Ferritin ≤200 ng/mL *N* = 2289	Ferritin >200 ng/mL *N* = 2140	*P* value
Age, years	49.5 ± 14.9	49.5 ± 14.7	49.4 ± 15.1	.741
Female, *n* (%)	1950 (44.0)	1142 (49.9)	808 (37.8)	<.001
CVD, *n* (%)	1417 (32.0)	727 (31.8)	690 (32.2)	.755
DM, *n* (%)	1242 (28.0)	670 (29.3)	572 (26.7)	.065
BMI, kg/m^2^	22.9 (3.5)	23.0 (3.7)	22.9 (3.4)	.604
SBP, mmHg	142 ± 20	142 ± 19	142 ± 20	.924
DBP, mmHg	87 ± 14	87 ± 13	87 ± 15	.865
Charlson comorbidity index	2 (2–4)	2 (2–4)	2 (2–4)	.424
Hemoglobin, g/L	103.5 ± 19.9	104.8 ± 19.6	102.1 ± 20.1	<.001
Serum albumin, g/L	35.51 ± 5.31	35.82 ± 5.02	35.17 ± 5.59	<.001
Serum creatinine, μmol/L	852 ± 293	821 ± 291	886 ± 292	<.001
Urea nitrogen, mmol/L	20.79 ± 7.57	20.45 ± 7.86	21.16 ± 7.24	.002
Total cholesterol, mmol/L	4.72 ± 1.21	4.81 ± 1.19	4.63 ± 1.21	<.001
Serum iPTH, pg/mL	286 (158–462)	287 (161–476)	284 (155–450)	.119
hsCRP, mg/L	2.61 (0.81–7.29)	2.28 (0.76–6.22)	3.06 (0.90–9.19)	<.001
TIBC, μmol/L	46.3 (15.4)	49.2 (17.8)	43.2 (11.6)	<.001
Ferritin, ng/mL	191.0(86.7–376.0)	88.8(47.5–137.3)	386.8 (275.8–586.8)	<.001
Serum iron, μmol/L	11.6 (8.8–15.2)	10.8 (8.1–14.0)	12.7 (9.6–16.5)	<.001
TSAT, %	25.9 (19.7–33.9)	23.2 (17.2–29.5)	29.6 (22.8–38.5)	<.001
Total *Kt*/*V* ≥1.7, *n* (%)	2315(67.3)	1320(72.2)	995(61.8)	<.001
Urine output, L/day	0.63 (0.20–1.15)	0.70 (0.28–1.20)	0.55 (0.16–1.10)	<.001
Iron supplementation, %	2327 (52.5)	1213 (53.0)	1114 (52.1)	.553
Oral iron supplementation, %	2209(49.9)	1192(52.1)	1017(47.5)	.003
IV iron supplementation, %	156(3.5)	44(1.9)	112(5.2)	<.001
Total elemental iron, mg/day	79 ± 67	83 ± 68	75 ± 66	.007
ESA administration, %	3372 (76.1)	1731 (75.6)	1641 (76.7)	.429
Epoetin dosage, U/kg/week	125 (86–167)	125 (88–164)	127 (85–172)	.186

A ferritin cut-off of 200 ng/mL was identified via RCS analysis as a key threshold for increased mortality risk.

RCS, restricted cubic spline; DM, diabetes mellitus; SBP, systolic blood pressure; DBP, diastolic blood pressure; iPTH, intact parathyroid hormone; TIBC, total iron-binding capacity; *Kt*/*V*, urea clearance.

Patients with serum ferritin levels exceeding 200 ng/mL exhibited lower Hb and serum albumin levels and higher levels of hsCRP compared with those with levels below 200 ng/mL. Furthermore, these patients demonstrated poorer residual kidney function, as evidenced by their daily urine output and total *Kt*/*V* (*P* < .001 for all), as presented in Table 1.

### Association between Hb and iron markers

**Figure 1:**
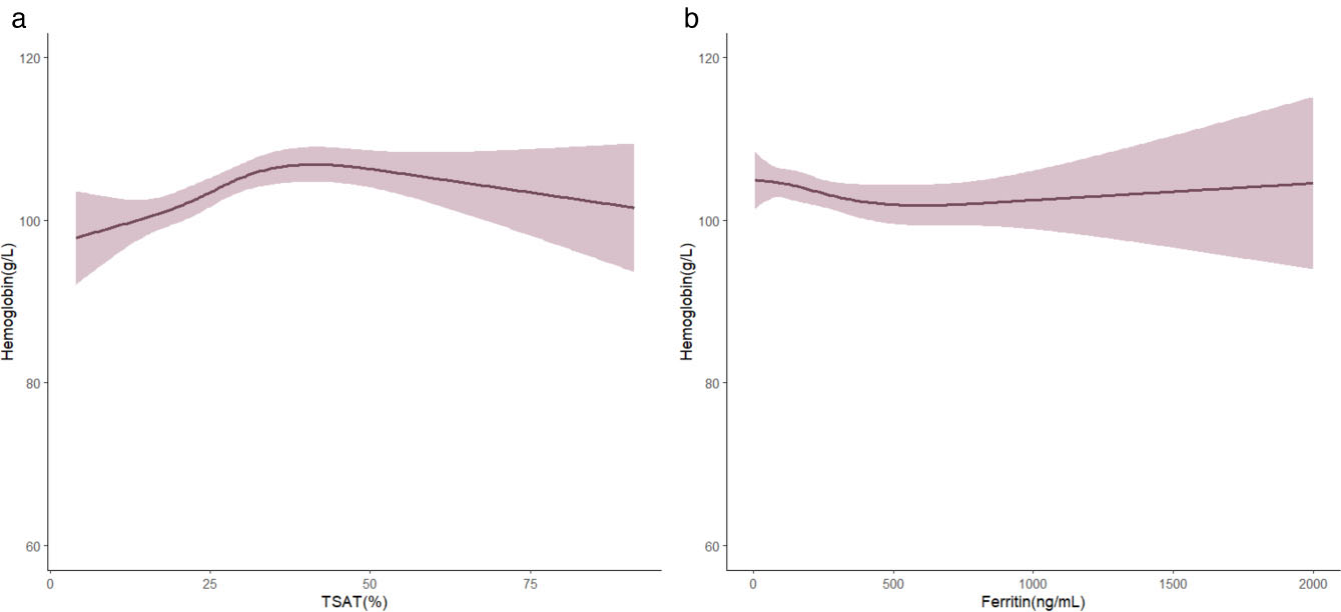
Association between TSAT, ferritin, and Hb. RCS plot of Hb by level of (**a**) TSAT and (**b**) ferritin. Models were adjusted for age, gender, Charlson score, BMI, serum albumin, iPTH, hsCRP, elemental iron and ESA doses.

We observed a non-linear association between Hb levels and both TSAT and ferritin. When TSAT was <40%, there was an increasing trend of Hb levels as TSAT rose; however, as TSAT continued to increase beyond this point, the rise in Hb levels tended to plateau and then gradually decreased. On the contrary, as ferritin levels increased, the concentration of Hb experienced a trend of decline until the ferritin level reached around 500 ng/mL, after which the trend of decrease in Hb began to stabilize (Fig. 1).

### Association between death and iron markers

**Figure 2:**
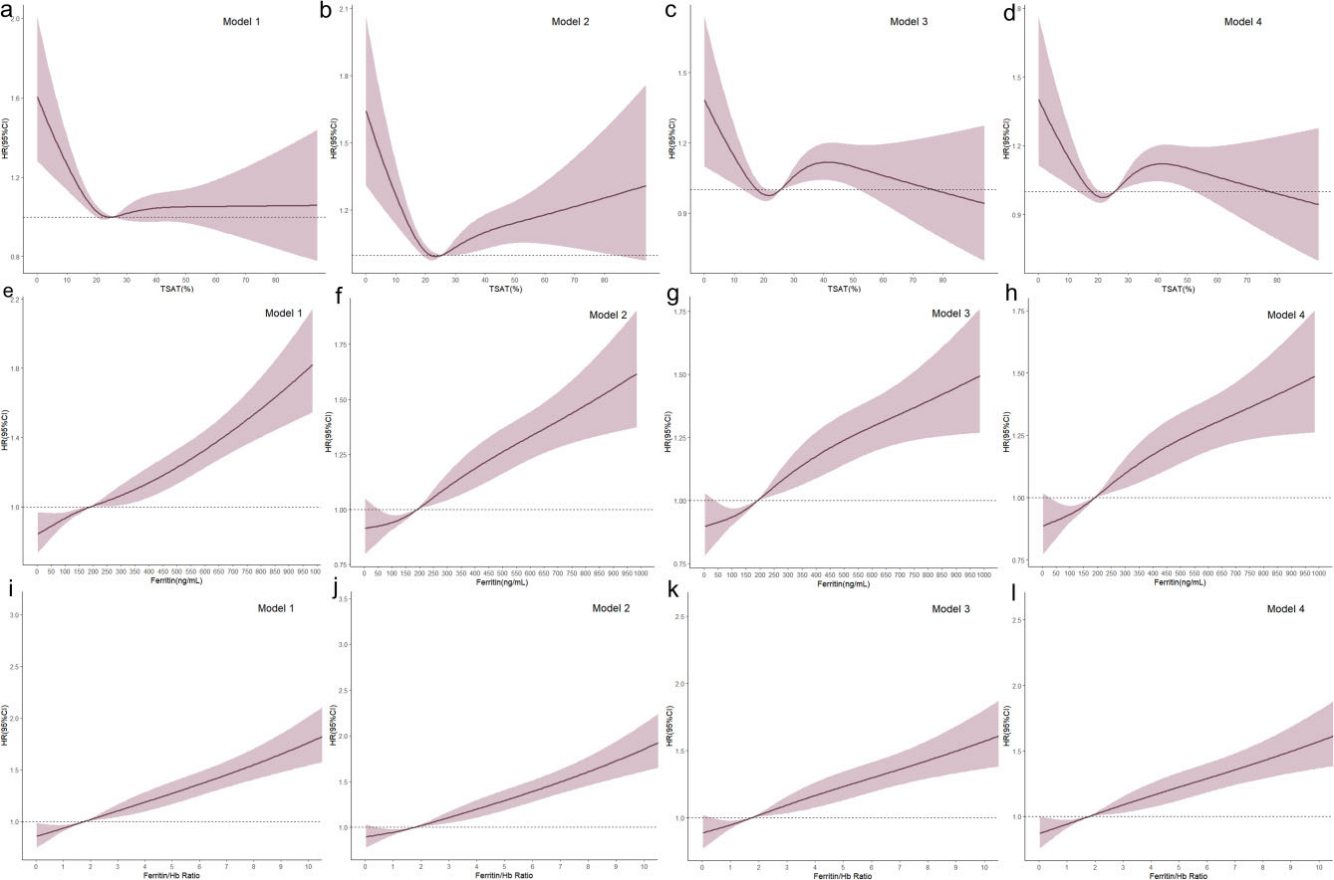
The association between iron markers and death. RCS plot of all cause death by levels of (**a–d**) TSAT (**e–h**) ferritin (**i–l**) Ferritin/Hb ratio. Model 1: unadjusted; Model 2: adjusted for age and gender; Model 3: Model 2 plus additionally adjusted for CVD history, serum albumin, serum total cholesterol and hsCRP; Model 4: Model 3 plus additionally adjusted for doses of elemental iron and ESA. TSAT, Transferring saturation; Hb, hemoglobin.

As shown in Fig. 2, we explored the associations of TSAT, ferritin levels, and the ferritin/Hb ratio with the mortality risk across four progressively adjusted models. In all four models (Fig. 2a–d) the relationship between TSAT and mortality risk showed a non-linear pattern. Our findings indicated a non-significant increase in mortality risk when TSAT levels fell below 20% (Fig. 2d). Ferritin levels exceeding 200 ng/mL were associated with a sharp increase in mortality risk, and this association remained consistent after adjusting for additional covariates in Models 2, 3, and 4. In Model 4, ferritin levels above 200 ng/mL were associated with a significantly elevated mortality risk (HR 1.207, 95% CI 1.134–1.286) (Fig. 2h). A similar trend was observed in the ferritin/Hb ratio; a ferritin/Hb ratio greater than 2 was significantly associated with an elevated mortality risk (HR 1.219, 95% CI 1.144–1.299) (Fig. 2l). To account for potential variability across different centers, we included center as a random effect in our analysis. This adjustment confirmed that the observed associations between ferritin levels, ferritin/Hb ratio, and mortality risk were consistent and not influenced by center-specific factors (Supplementary Fig. S2).

### Impact of inflammation on the association between ferritin, ferritin/Hb ratio, and mortality

**Figure 3:**
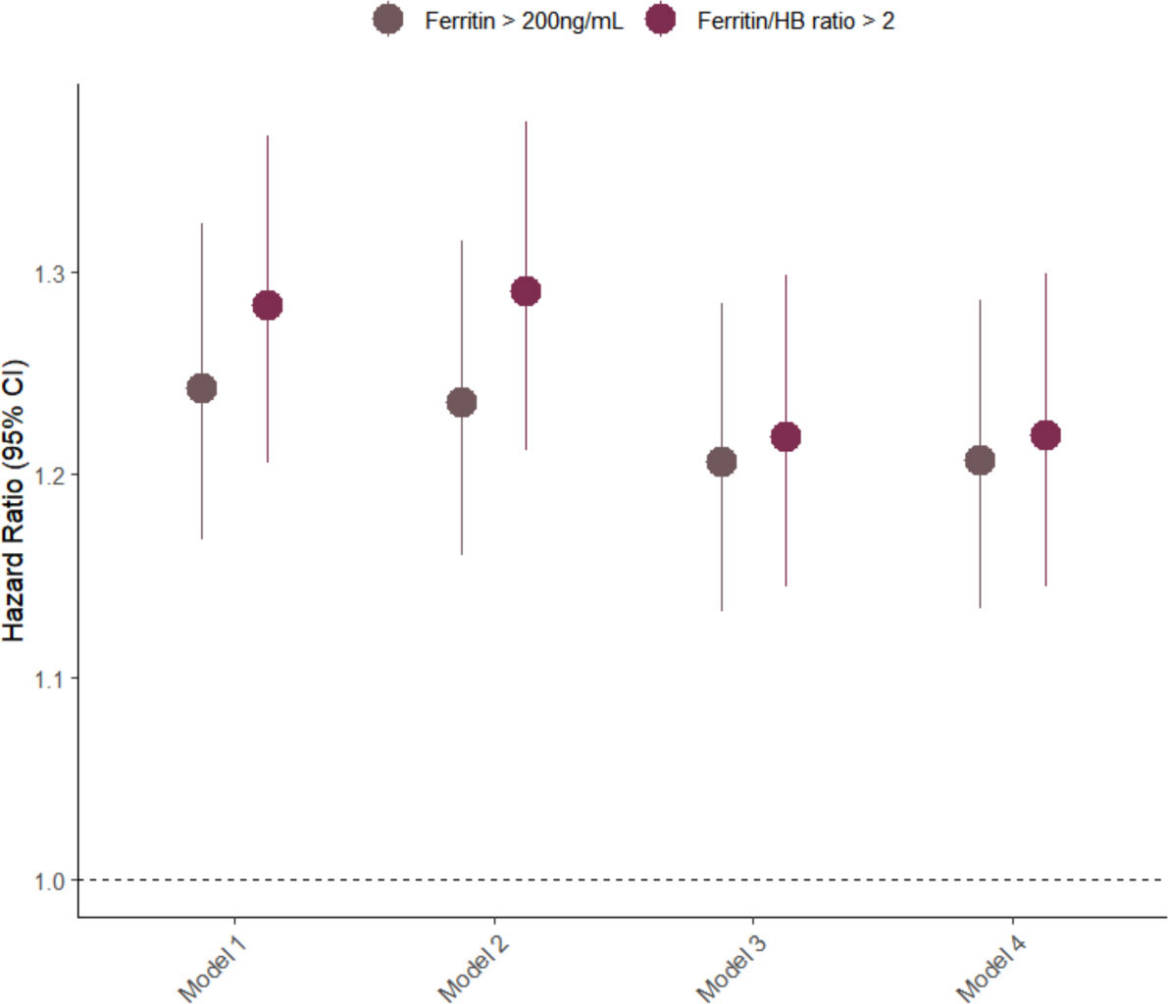
Interaction between ferritin >200 ng/mL, Ferritin/Hb ratio >2, and hsCRP tertiles on mortality risk. Forest plot showing the HRs for all-cause mortality by levels of (**a**) ferritin >200 ng/mL and (**b**) ferritin/hemoglobin ratio >2 across hsCRP tertiles. Models were adjusted for age, gender, CVD history, serum albumin, serum total cholesterol, hsCRP, and doses of elemental iron and ESA.

We further explored the interaction between inflammation, as indicated by hsCRP tertiles, and the association of ferritin >200 ng/mL and ferritin/Hb ratio >2 with mortality. Overall, the risk of mortality associated with both ferritin >200 ng/mL and ferritin/Hb ratio >2 decreased as hsCRP levels increased. In the first hsCRP tertile, both ferritin >200 ng/mL and ferritin/Hb ratio >2 were significantly associated with an increased risk of mortality, with hazard ratios of 1.297 (95% CI 1.134–1.484) and 1.426 (95% CI 1.246–1.631), respectively. In the second tertile, the associations were slightly attenuated: ferritin >200 ng/mL had an HR of 1.203 (95% CI 1.067–1.357) and ferritin/Hb ratio >2 had an HR of 1.245 (95% CI 1.104–1.404). By the third tertile (highest inflammation), the associations were further weakened, with ferritin >200 ng/mL showing an HR of 1.088 (95% CI 0.995–1.190) and ferritin/Hb ratio >2 showing an HR of 1.056 (95% CI 0.965–1.155) (Fig. 3).

### Association between death and iron markers across different subgroups

**Figure 4:**
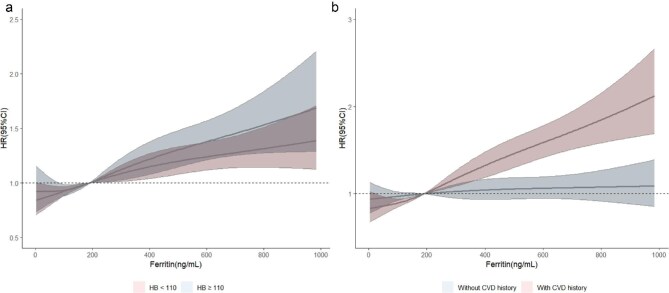
The association between ferritin and death across different subgroups. Predicted death by ferritin in (**a**). hemoglobin <110g/L or ≥110g/L, (**b**). CVD and non-CVD group. Models were adjusted for age, gender, CVD history, serum albumin, serum total cholesterol, hsCRP and doses of elemental iron and ESA. HB, hemoglobin, CVD, cardiovascular disease.

For both patients with Hb <110 g/L and those with Hb ≥110 g/L, ferritin levels exceeding 200 ng/mL were linked to a significant increase in mortality risk. However, the risk was more pronounced in patients with Hb <110 g/L, where the risk increased sharply as ferritin levels rose. In patients with Hb ≥110 g/L the increase in mortality risk was more gradual, but still evident (Fig. 4a). In contrast, when stratified by CVD history, the association between ferritin and mortality risk differed markedly. In patients with a history of CVD, ferritin levels above 200 ng/mL were associated with a substantial increase in mortality risk, with the HR rising significantly as ferritin levels increased. However, in patients without a history of CVD, ferritin levels above 200 ng/mL did not significantly increase mortality risk (Fig. 4b).

**Figure 5:**
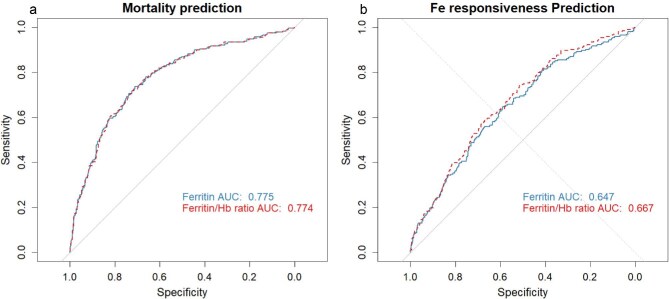
ROC curves comparing the predictive performance of ferritin and the ferritin/Hb ratio for (**a**) mortality and (**b**) iron responsiveness.

### Comparison of predictive performance of ferritin and the ferritin/Hb ratio for mortality and iron responsiveness

The ROC curve analysis showed that for mortality prediction the AUC was 0.775 for ferritin and 0.774 for the ferritin/Hb ratio, with no significant difference (*P* = .901). For predicting responsiveness to iron therapy, the ferritin/Hb ratio demonstrated a significantly higher AUC of 0.667 compared with 0.647 for ferritin alone (*P* < .001).

## DISCUSSION

In this multicenter prospective cohort study involving 4429 PD patients, we observed that Hb levels increased with rising TSAT up to a threshold of 40%, beyond which no further increases were noted. Conversely, an elevation in serum ferritin levels was associated with a decline in Hb concentrations. Notably, ferritin levels exceeding 200 ng/mL were linked to a 20.7% increase in mortality risk, independent of other potential confounders. This association was particularly pronounced in patients with a prior history of CVD and low hsCRP levels. Furthermore, a ferritin/Hb ratio greater than 2 was identified as an independent predictor of mortality risk within this PD population.

Our PD cohort has indicated that Hb level less than 110 g/L is associated with increased risk of mortality [21]. To date, there is a lack of robust data to support specific target values for iron metabolism markers for initiation or termination to guide iron treatment in patients undergoing PD. The Kidney Disease Outcomes Quality Initiative (KDOQI) guidelines state that there is insufficient evidence to recommend routine administration of intravenous iron if the serum ferritin level is greater than 500 ng/mL [8]. Similarly, the KDIGO guideline suggested a trial of intravenous iron while TSAT is ≤30% and ferritin is ≤500 ng/mL [5]. Our study demonstrated that Hb levels elevated with the increase of TSAT until 40%, followed with a mild decrease afterwards. This trend was consistent with the observations in the HD population [22]. In addition, a TSAT level lower than 20% seemed to be associated with a trend of increased mortality in our cohort. Taking into account that TSAT less than 20% was suggested to be representative of circulating iron deficiency [23, 24], we considered that 20%–40% TSAT might be an appropriate range for iron supplementation in the PD population.

In the literature, studies on the association between ferritin and mortality in the PD population were limited. Only the study conducted by Maruyama *et al*. compared the association between higher serum ferritin values and mortality in PD and HD patients but only found positive results in HD patients [25]. Our data suggested that serum ferritin levels exceeding 200 ng/mL were associated with a 20.7% increased risk of mortality, and this effect remained significant after adjusting for demographic and nutritional factors (serum albumin, total cholesterol, and hsCRP), iron supplements and erythropoiesis agents. This finding, while contradicting some hemodialysis studies, such as the pivotal study [26] demonstrating that maintaining ferritin levels around 700 ng/mL with intravenous iron reduced mortality risk compared with strategies aimed at preventing levels from dropping below 200 ng/mL, can be attributed to several factors. Firstly, our cohort consists of Chinese PD patients, whose genetic, dietary, and environmental influences on iron metabolism differ from those of Western HD populations. These differences may lead to varying sensitivities to ferritin levels, potentially explaining the lower risk threshold observed. As demonstrated by the DOPPS findings, the threshold at which elevated ferritin levels pose a mortality risk in Japanese HD patients is significantly lower compared with that observed in HD patients from Western countries [16]. Additionally, patients with higher ferritin levels in our cohort also exhibited elevated hsCRP levels, suggesting an inflammatory state. This can elevate ferritin levels independently of iron stores and correlate with adverse outcomes, even at levels considered safe by KDIGO guidelines. Furthermore, PD patients experience distinct fluid and solute removal dynamics and undergo less frequent blood monitoring compared with HD patients. Ferritin is not only a marker of iron stores but also an acute-phase reactant that increases in response to inflammation. In the literature, higher serum ferritin has been proven to be associated with EPO resistance, malnutrition, and inflammation in dialysis patients, which could to a great extent explain the negative association between ferritin and mortality in our PD subjects and HD patients in previous literature [16, 27]. Notably, the risk cut-off value identified in the current study is indeed lower than the thresholds reported in several previous studies [13–15]. It is noteworthy that the proportion of patients with *Kt*/*V* >1.7 was statistically different between the two ferritin cut-off groups. While there is no direct evidence linking inadequate dialysis to elevated ferritin levels, inadequate dialysis can lead to chronic inflammation, which may in turn contribute to increased ferritin levels.

Notably, the average Hb levels in our cohort did not meet the levels recommended by the KDIGO guidelines and were lower compared with the Hb levels of patients in national populations studied in the PDOPPS research [12]. Results from DOPPS and PDOPPS on iron metabolism markers in dialysis patients showed significant variation across different ethnic groups, and there were also differences in the approaches to interventions using iron supplements and ESAs [12, 16]. The median elemental iron supplement was 68.0 (27.5–125) mg/day in our cohort. Only 49.9% of patients were treated with oral iron supplements, and 3.5% received intravenous iron. This may reflect a common incompliance with oral supplements in PD patients or insufficient prescription by clinicians. The inconvenience of access to intravenous iron supplements also potentially contributes to the low iron doses. It cannot be excluded that the Chinese PD population may benefit from a more proactive approach to iron supplementation. However, our data also showed no extra benefits on Hb while serum ferritin increased (Fig. 1) and a negative association between ferritin and mortality, as described above. Taking the results together, we need to balance the benefits and harms of iron supplements by monitoring ferritin and TSAT levels individually. Novel agents facilitating iron utilization, such as hypoxia-inducible factor (HIF) prolyl hydroxylase inhibitors, which enhance iron utilization by stabilizing HIF and increasing the expression of genes involved in iron transport and absorption, are desired [28, 29].

We further found for the first time that a ferritin/Hb ratio higher than 2 was associated with increased risk of mortality in the PD population. The ROC curve analysis highlights the clinical utility of the ferritin/Hb ratio. While both ferritin and the ferritin/Hb ratio showed similar predictive power for mortality, the ferritin/Hb ratio demonstrated superior performance in predicting responsiveness to iron therapy. In clinical practice, the EPO/Hb ratio has been commonly utilized to gauge a patient's responsiveness to ESA therapy. A higher ESA resistance index suggests that a patient is less responsive to ESA, guiding physicians to avoid unnecessary increases in ESA dosage, which could lead to adverse effects without improving anemia [30, 31]. Similarly, a higher ferritin/Hb ratio might indicate sufficient or excessive iron stores relative to Hb production, suggesting that further iron supplementation could be ineffectual or even harmful. Conversely, a lower ferritin/Hb ratio might signal inadequate iron stores, necessitating an increase in iron supplementation to support erythropoiesis effectively. By monitoring the ferritin/Hb ratio, healthcare providers may make more informed decisions about iron supplementation, potentially reducing the risks associated with both iron deficiency and overload based on individual conditions. The benefits of monitoring ferritin/Hb ratio require further validation through high-quality and large-scale studies in the PD population.

This study's advantage is derived from its detailed exploration of the implications of iron metabolism markers for the management of anemia and prognostic outcomes in PD patients, leveraging data from a large sample population. In our analysis, we meticulously evaluated the potential influences of varying dosages of iron supplements and ESAs on the study outcomes. We have introduced the ferritin/Hb ratio as a novel tool to aid clinicians in evaluating the relationship between iron supplementation and anemia. This indicator independently predicted patient outcomes. Our study has limitations. As it was an observational study, we could not completely rule out all the residual confounders. Specifically, factors such as smoking and chronic alcohol consumption, which are known to increase ferritin levels, were not fully accounted for in our analysis. Additionally, while we attempted to minimize the impact of acute infections by averaging baseline data over 3 months, this method may not have fully excluded all patients experiencing transient infections. This could potentially have influenced our results. Furthermore, all of the patients enrolled from the PDTAP were Chinese patients. The normal values for indicators of iron metabolism in the Asian dialysis population may differ from those in Western populations. Our study included patients over 14 years of age, differing from the European Union standard, where adults are typically over 18. This choice reflects our study population and healthcare setting but may limit external validity, especially compared with registries like the European Renal Association, where the mean age is higher. These differences should be considered when assessing the generalizability of our results.

## CONCLUSIONS

Through this multicenter prospective cohort, we observed that a serum ferritin level exceeding 200 ng/mL was associated with a higher risk of mortality, independent of well-recognized confounders in the PD population. This study further suggested that ferritin should receive greater consideration as a significant surrogate of worse outcomes among patients having a CVD history or those without obvious hsCRP elevation. We report for the first time that a ferritin/Hb ratio greater than 2 was an independent predictor of mortality risk. Whether monitoring the ferritin/Hb ratio would be helpful in reducing the risks associated with both iron deficiency and overload individually needs to be explored further.

## Supplementary Material

sfae427_Supplemental_Files

## Data Availability

Due to restrictions imposed by the Chinese Human Genetic Resources Administration, the raw data from this study cannot be shared. However, under specific circumstances, secondary analyses of the data may be conducted by contacting the corresponding author.
