# Supplementary material for: Associations between iron markers with hemoglobin and outcomes in peritoneal dialysis patients: results from the PDTAP study
Source: Clin Kidney J. 2024 Dec 30;18(4):sfae427. doi: 10.1093/ckj/sfae427 (PMC12032523; doi:10.1093/ckj/sfae427)
Supplement: sfae427_Supplemental_Files [file sfae427_supplemental_files.zip › 793 Supplemental Table 1.docx]

**Supplemental Table 1| Additional characteristics of the study subjects.**

|  | Total population  N= 4429 | Ferritin≤200ng/mL  N=2289 | Ferritin>200ng/mL  N=2140 | *P* value |
| --- | --- | --- | --- | --- |
| Blood glucose, mmol/L | 6.27±4.15 | 6.33±4.40 | 6.22±3.86 | 0.382 |
| Triglycerides, mmol/L | 1.48 (1.10-2.13) | 1.44 (1.07-2.03) | 1.55 (1.14-2.21) | <0.001 |
| Serum HDL, mmol/L | 1.17±0.42 | 1.22±0.43 | 1.12±0.41 | <0.001 |
| Serum LDL, mmol/L | 2.74±0.86 | 2.80±0.87 | 2.67±0.85 | <0.001 |
| Serum calcium, mmol/L | 2.18±0.24 | 2.17±0.23 | 2.19±0.24 | 0.025 |
| Serum phosphorus, mmol/L | 1.64±0.62 | 1.64±0.75 | 1.64±0.45 | 0.851 |
| Total Kt/V | 1.89 (1.60-2.25) | 1.97 (1.67-2.33) | 1.82 (1.53-2.15) | <0.001 |
| Renal Kt/V | 0.41 (0.07-0.83) | 0.50 (0.14-0.91) | 0.30(0.00-0.72) | <0.001 |
| Peritoneal Kt/V | 1.42 (1.12-1.74) | 1.44 (1.14-1.74) | 1.40 (1.09-1.73) | 0.096 |
| Total CCr, L/w/1.73 m^2^ | 56.68 (46.61-72.10) | 57.84 (47.51-74.43) | 55.17 (45.70-69.77) | <0.001 |
| Renal CCr, L/w/1.73 m^2^ | 14.70 (2.15-33.26) | 17.52 (4.50-36.49) | 11.38 (0.00-28.79) | <0.001 |
| Peritoneal CCr, L/w/1.73 m^2^ | 40.43 (31.83-47.44) | 39.90 (30.52-47.24) | 40.95 (33.49-47.66) | 0.001 |

A ferritin cut-off of 200 ng/mL was identified via RCS analysis as a key threshold for increased mortality risk.

Abbreviations: RCS, restricted cubic spline; LDL, low density lipoprotein; HDL, high density lipoprotein; Total CCr, total creatinine clearance; Total Kt/V, total urea clearance.
