# Supplementary figures and images for: Associations between iron markers with hemoglobin and outcomes in peritoneal dialysis patients: results from the PDTAP study
Source: Clin Kidney J. 2024 Dec 30;18(4):sfae427. doi: 10.1093/ckj/sfae427 (PMC12032523; doi:10.1093/ckj/sfae427)

## Slide 1
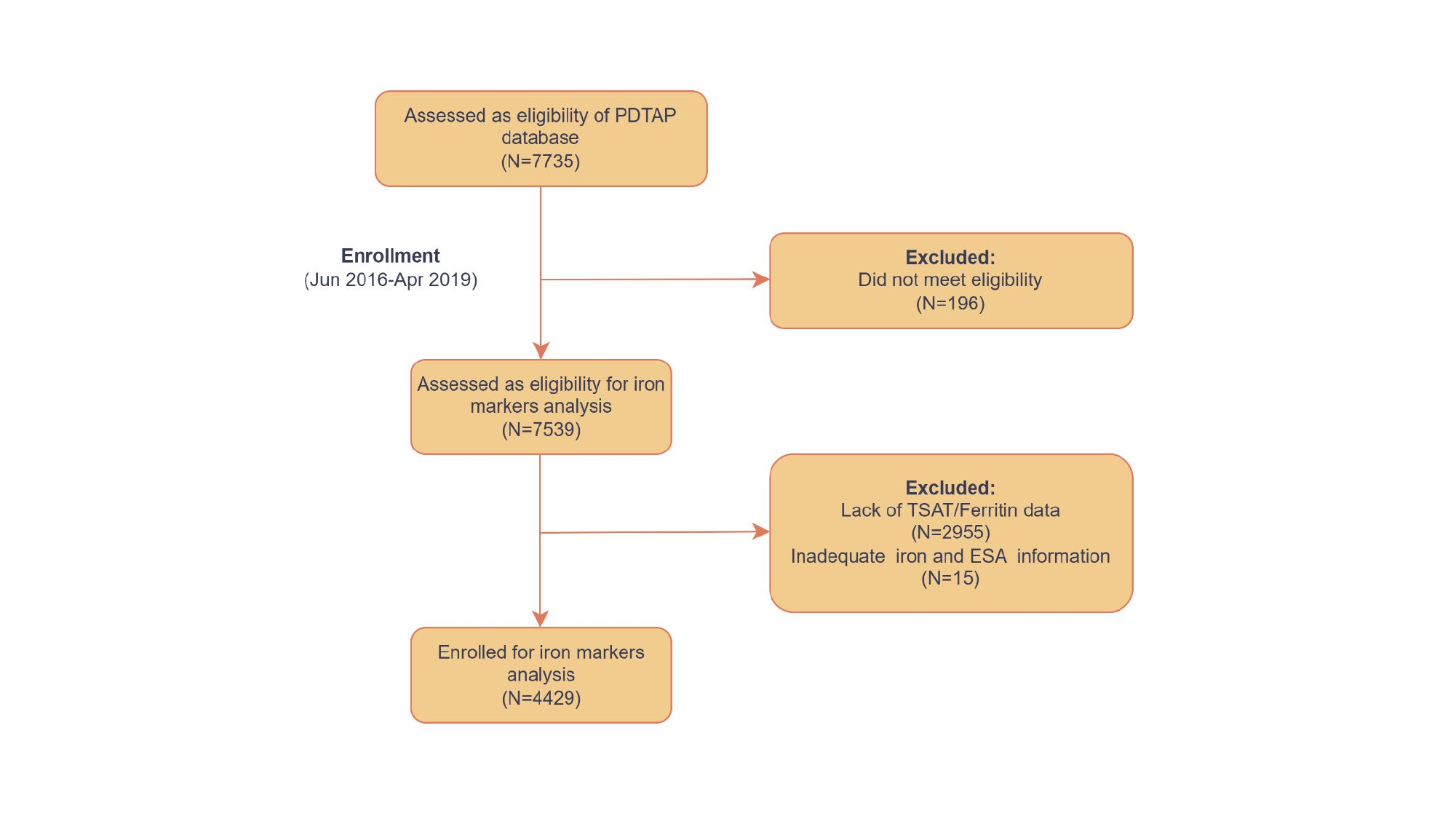

Supplement: sfae427_Supplemental_Files [file sfae427_supplemental_files.zip › Supplemental Figure 1.pptx]

## Slide 1
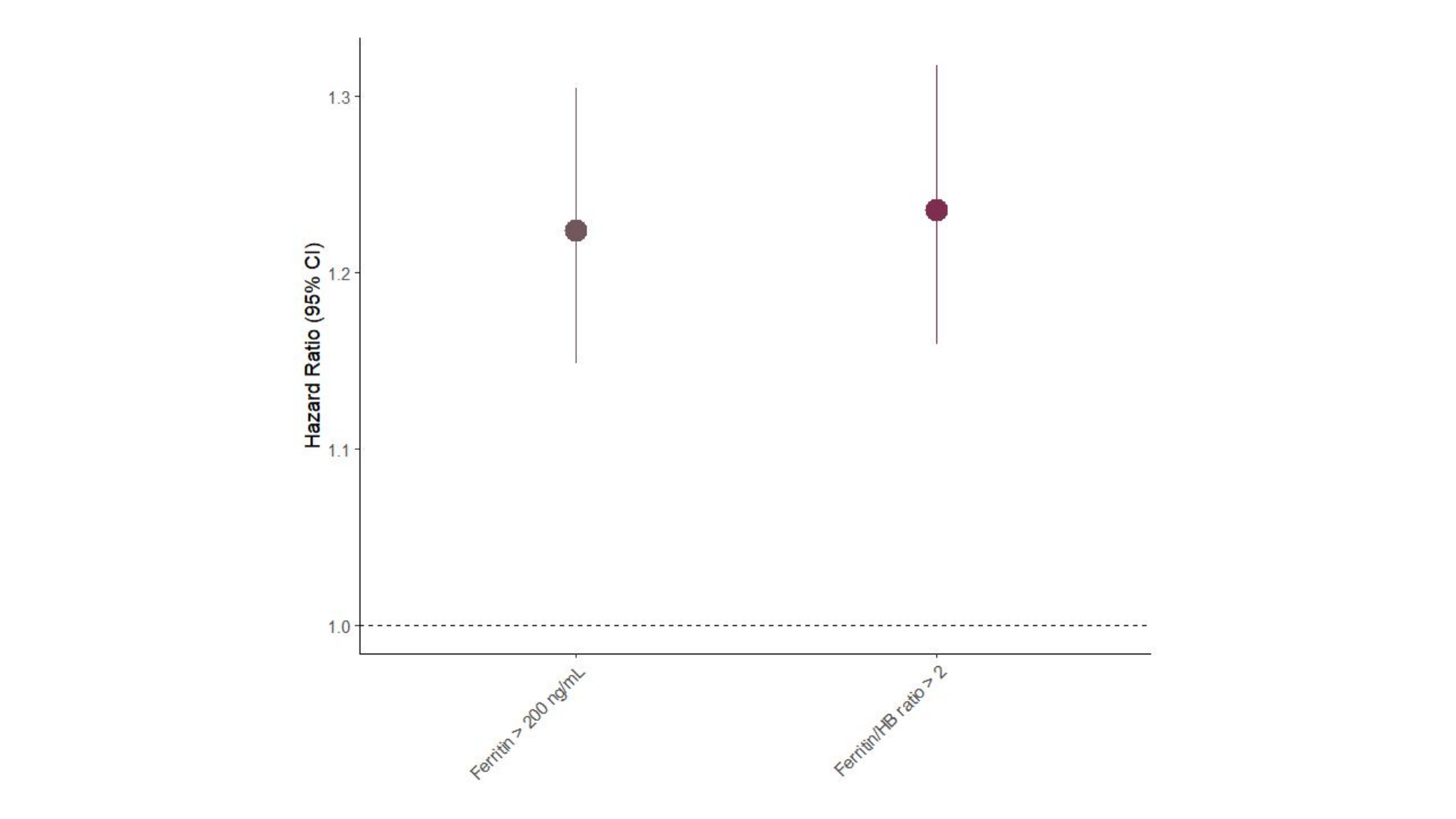

Supplement: sfae427_Supplemental_Files [file sfae427_supplemental_files.zip › Supplemental Figure 2.pptx]

## Slide 1
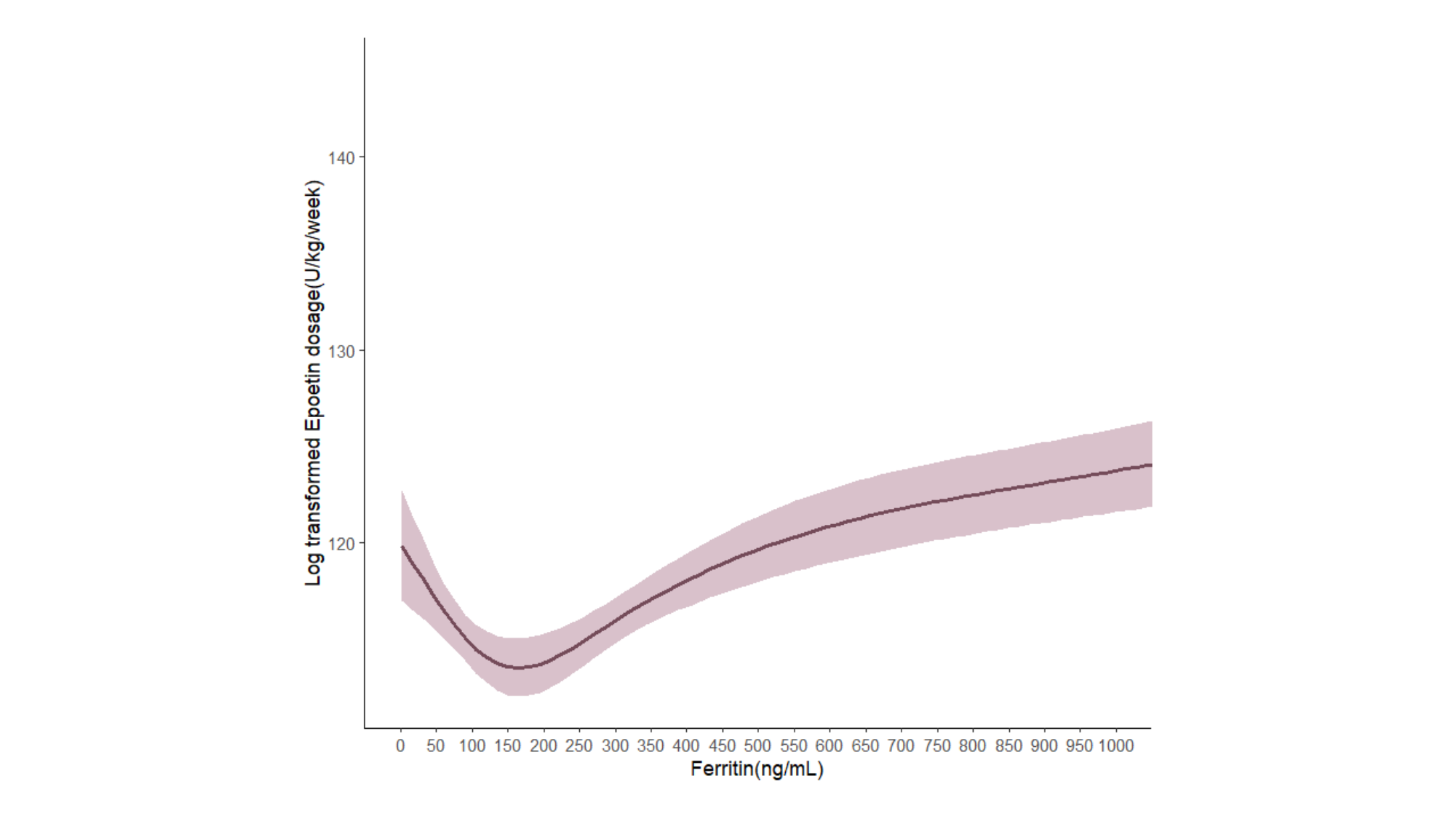

Supplement: sfae427_Supplemental_Files [file sfae427_supplemental_files.zip › Supplemental Figure 3.pptx]
